# Implant stability in patients treated with platelet‐rich fibrin and bovine bone substitute for alveolar ridge preservation is associated with peripheral blood cells and coagulation factors

**DOI:** 10.1002/cre2.263

**Published:** 2019-12-06

**Authors:** Joost E.I.G. Brouwers, Lisa N. van der Vorm, Sharon Buis, Rianne Haumann, Avesta Karanzai, Joke Konings, Philip G. de Groot, Bas de Laat, Jasper A. Remijn

**Affiliations:** ^1^ Institute for Dental Implantology Amersfoort The Netherlands; ^2^ Department of Clinical Chemistry and Hematology Gelre Hospitals Apeldoorn The Netherlands; ^3^ Synapse Research Institute Maastricht The Netherlands; ^4^ Cardiovascular Research Institute Maastricht Maastricht University Medical Centre Maastricht The Netherlands; ^5^ Department of Clinical Chemistry Meander Medical Center Amersfoort The Netherlands

**Keywords:** alveolar ridge preservation, blood cells, blood coagulation, implant stability, platelet‐rich fibrin

## Abstract

**Aims:**

The aim of the present study was to assess the association between dental implant stability and peripheral blood cell composition and levels of coagulation factors in patients treated with alveolar ridge preservation with platelet‐rich fibrin (PRF) and bovine bone substitute.

**Materials and methods:**

Fifty patients were included between 2015 and 2017. PRF was prepared from autologous blood, in which blood cells and coagulation factor levels were measured. PRF and bovine bone were placed in the socket, followed by closure with PRF membrane. Implants were placed 14 (±2.5) weeks postextraction. The implant stability quotient was measured at *t* = 0, *t* = 10 days, *t* = 7 weeks, and *t* = 17 weeks by resonance frequency analysis.

**Results:**

Erythrocyte count was inversely associated with PRF membrane length, but not with implant stability. Conversely, platelet count did not correlate with membrane size but inversely correlated with implant stability at 7 and 17 weeks. In addition, implant stability was directly correlated with levels FXIII (*t* = 0, *p* < .01), active von Willebrand factor (VWF; *t* = 0 and 7 weeks, *p* < .05), and total VWF (*t* = 7 weeks, *p* = .012).

**Conclusion:**

Implant stability following alveolar ridge preservation with PRF and bovine bone substitute is associated with circulating blood cells and coagulation factors. In particular, fibrin structure, VWF, and FXIII may be important modulators of implant stability.

## 1 INTRODUCTION

Upon tooth extraction or tooth loss, progressive resorption of the alveolar bone occurs (Araujo & Lindhe, 2005; Chappuis et al., 2013). This can lead to insufficient height and width of the residual bone, which negatively influences the prognosis of implant‐supported prostheses (Ashman, 2000; Esposito, Grusovin, Worthington, & Coulthard, 2006). One of the available treatment options to prevent this is alveolar ridge preservation (Darby, Chen, & Buser, 2009; Wang & Lang, 2012).

Platelet‐inspired biomaterials have been extensively studied for this purpose. In particular, second‐generation platelet concentrates, such as leukocyte‐ and platelet‐rich fibrin (PRF; Choukroun, 2001; Dohan et al., 2006a), have important advantages: preparation of PRF is relatively fast, easy, and no additives or anticoagulants are required, which makes PRF more economic than other platelet‐dependent biomaterials used to fill the alveole (Dohan Ehrenfest, Rasmusson, & Albrektsson, 2009). PRF consists of a slowly polymerized fibrin network with enmeshed cytokines, glycanic chains, structural glycoproteins, platelets, and leukocytes (Dohan et al., 2006b; Dohan Ehrenfest et al., 2009). Platelets secrete fibrinogen, fibronectin, and vitronectin, which behave as a matrix for connective tissue and adhesion molecules, facilitating cell recruitment to the wound area (Assoian, Komoriya, Meyers, Miller, & Sporn, 1983; Banks et al., 1998; De Pascale, Sommese, Casamassimi, & Napoli, 2015; Lacci & Dardik, 2010; Mehta & Watson, 2008). The fibrin network has a key role in early wound healing and functions as a scaffold for ingrowing cells and as a reservoir of cytokines (Soloviev et al., 2014; van Hinsbergh, Collen, & Koolwijk, 2001). Importantly, the cells entrapped in the fibrin mesh release a variety of growth factors (Dohan et al., 2006b; Peterson et al., 2010; Schar, Diaz‐Romero, Kohl, Zumstein, & Nesic, 2015).

The use of PRF (with or without additional bone grafting material, such as deproteinized bovine bone mineral, DBBM) for ridge preservation has been reported previously. However, its efficacy has been evaluated with heterogeneous approaches, and results are inconsistent (Castro et al., 2017; Pan et al., 2019; Temmerman et al., 2016). Interestingly, it was observed that some patients have smaller and/or shorter PRF membranes than others (Mazzocca et al., 2012). This may in part be due to differences in red blood cell (RBC) content, as individuals with lower hematocrit have larger PRF membranes (Mazzocca et al., 2012; Miron et al., 2019). Indeed, the composition of the peripheral blood, including but not limited to RBC content, might influence the size and composition of PRF. Besides the cellular fraction, circulating coagulation factors may also influence the properties of the PRF and hence implant stability.

Therefore, in the current study, we investigated the association between implant stability and the peripheral blood cell composition and levels of coagulation factors closely involved in fibrin network formation and platelet incorporation.

## 2 MATERIALS AND METHODS

### 2.1 Ethics statement

The research complied with all the relevant national regulations, institutional policies, and the tenets of the Helsinki Declaration (2013) and has been approved by the Medical Ethical Review Board of the Gelre Hospitals Apeldoorn (Reference 15.38; 28‐07‐2015). All participants gave full written informed consent.

### 2.2 Study population

This study was designed as a prospective observational study. All study participants were enrolled from the Institute of Oral Implantology (Amersfoort, the Netherlands) between September 2015 and September 2017. We included patients with age ≥ 18 years, requiring an implant in posterior maxilla or mandible, and classified as American Society of Anaesthesiologists I (healthy individual) or American Society of Anaesthesiologists II (mild systemic disease) patients. Six of our patients were current smokers, with an average smoking quantity of six cigarettes per day (*SD* = 3). Three patients were suffering from high blood pressure, treated with an angiotensin‐converting enzyme inhibitor or angiotensin 2 receptor blocker. Five patients had a history of respiratory illness (i.e., asthma and bronchitis), and two of them were treated with either a sympathomimetic or inhalation steroids. One patient had a previous myocardial infarction (2007) and was therefore treated with aspirin and statins.

### 2.3 Preparation of PRF membranes

Three to four (depending on the implant site) 10‐ml A‐PRF tubes (Process for PRF, Nice, France) were obtained by venipuncture prior to the atraumatic extraction procedure. The A‐PRF tubes were immediately centrifuged (PRF DUO, Process for PRF, Nice, France) at an RCF of 200 *g* (calculated using the formula RCF = 1.12 × Radius × (rpm/1,000)2, based on rpm of 1,336 and rotor radius 100 mm), for 8 min. After centrifugation, the tubes were left in an upright position for 10 min. Three layers were obtained, namely, RBCs at the bottom, platelet‐poor plasma at the top, and PRF in between. PRF membranes were separated from the red thrombus and placed in the metal PRF box (Process for PRF, Nice, France). Subsequently, PRF exudate was obtained using the lid of the PRF box as weight. PRF membrane length and width (both in cm) were measured. Subsequently, one of the membranes was cut into fragments and mixed with DBBM (Bego‐Oss®, approximately 0.5–1 ml) and the growth factor‐rich PRF exudate. The other PRF membranes were used to close the socket.

### 2.4 PRF treatment protocol

The study procedure consisted of two stages, namely, ridge preservation with PRF and implant placement, as described below. All procedures were performed under local anesthesia by the same implantologist (J. B.). Atraumatic extraction was performed to preserve as much bone as possible and avoid fracture of the buccal plate. Periotomes and small luxators were used to remove the teeth. DBBM (on average 0.5–1 ml, Bego‐Oss®, BEGO Implant Systems GmbH & Co., Bremen, Germany), on average 0.5–1 ml, mixed with PRF (see Section 2.3) was placed into the socket until the desired vertical height was achieved. An envelope flap was created on the buccal and palatal side. A PRF membrane was placed in the envelope flap, followed by a mattress suture to fixate the membrane. No antibiotics or pain medications were prescribed before extraction and postoperatively. After a healing period of 14 ± 2.5 weeks, the implant site was prepared using a trephine drill with a diameter of 2 mm. Bone level implants (Bego RS or SC implants, BEGO) with diameters between 4.1 and 5.5 mm were placed. Insertion torque (N/cm) was determined using the Oral Implantcenter (Acteon, Bordeaux, France). All patients were followed 17 weeks after implantation.

### 2.5 Analysis of blood cell and coagulation parameters

Peripheral blood samples were obtained by venipuncture (concurrently with blood for PRF preparation, prior to extraction) into two 2‐ml EDTA tubes and two 2‐ml 3.2% sodium citrate tubes (Vacuette, Greiner Bio‐One, Kermsmunster, Austria). Blood cell counts (platelets, erythrocytes, hematocrit, and leukocytes) were measured using the hematology Cell‐Dyn Sapphire analyzer (Abbot Diagnostics, Wiesbaden, Germany). Clotting times (PT and APTT) and the coagulation factors fibrinogen, FVIII and FXIII, were measured using the STA‐Max analyzer (Stago, Asnières‐sur‐Seine, France). To measure von Willebrand factor (VWF) and active VWF, that is, VWF in its unfolded state able to bind platelets via the GPIb platelet receptor, we used in‐house sandwich enzyme‐linked immunosorbent assays, as described earlier (van der Vorm et al., 2019). (Active) VWF levels are expressed as a percentage (%) of levels measured in normal pooled plasma.

### 2.6 Measurement of implant stability

The stability of the implants was evaluated with resonance frequency analysis. The measurements were carried out with the Osstell device (Osstell, Göteborg, Sweden). The implant stability quotient (ISQ) has a range from 1 to 100, with a higher number indicating a more stable implant. ISQs were measured buccal/pallatinal (bp) and mesial/distal (md). Ideally, the same ISQ value is found from both directions, indicating that the bone‐implant interface is the same around the implant. However, if the bone is inhomogeneous, the implant can have different stability in different directions. Therefore, we provide both the bp and md ISQ values. ISQ measurements were performed immediately after surgery (*t* = 0, *n* = 38) and 10 days (*n* = 36), 7 weeks (*n* = 39), and Week 17 (*n* = 50) after implant placement.

### 2.7 Statistical analysis

The normality of continuous variables was assessed graphically in histograms and normality Q–Q plots and tested using the Shapiro–Wilk test. Continuous variables with a skewed distribution are presented as median (interquartile range [IQR]). Values for continuous nonskewed variables are presented as means ± SD. Correlations between parameters are expressed as the Spearman's rank correlation coefficient *r*, with the corresponding *p* value. Comparison of the clinical outcome parameter between different time points was performed by Kruskal–Wallis test with pairwise post hoc test (including Bonferroni correction for multiple comparisons). IBM Statistical Package for Social Sciences Version 25 software was used for all statistical analyses (SPSS Incorporated, Chicago, USA). Figures were prepared using GraphPad Prism version 5.00 (GraphPad Software, San Diego, USA). A *p* value of <.05 was considered statistically significant.

## 3 RESULTS

### 3.1 Patient characteristics and implant stability measurements

Patient demographics are provided in Table 1. The study consisted of 50 patients (28 males [56%] and 22 females [44%]) with an age range between 35 and 82 years (mean 58.8 ± *SD* 11.2 years), treated with a composite graft of PRF and DBBM for alveolar ridge preservation prior to placement of a dental implant.

**Table 1:** Patient demographics and clinical data on implant stability

Variable	Mean ± *SD* or median (IQR)	N
Age, years	58.8 ± 11.2	50
Sex (% male)	56	50
Current smoker (%)	12	50
Membrane and implant		
Length (cm)	3.1 ± 0.4	41
Width (cm)	1.2 (0.3)	17
Area (cm^2^)	3.9 ± 0.9	17
Insertion torque (*N*/cm)	50 (18)	44
Maxillary implant^a^	62%	31
Total nr. of extractions	62	50
Molars extracted (*n*)	37	50
ISQs		
*t* = 0 bp	73.5 (5)	38
*t* = 0 md	74.5 (5)	38
*t* = 10 days bp	78.5 ± 5.4	28
*t* = 10 days md	79.6 ± 4.3	28
*t* = 7 weeks bp	77.0 (7.0)	30
*t* = 7 weeks md	79.0 (5.0)	30
*t* = 17 weeks bp	79.0 (9.0)	50
*t* = 17 weeks md	81.5 (8.0)	50

Abbreviations: bp, buccal–palatal; ISQ, implant stability quotient; md, mesial–distal.

Out of the 50 implants in total, 19 (38%) were placed in the mandibula and 31 (62%) in the maxilla. *N* in the last column indicates the total number of patients from which data on each specific parameter were available.

**Figure 1:**
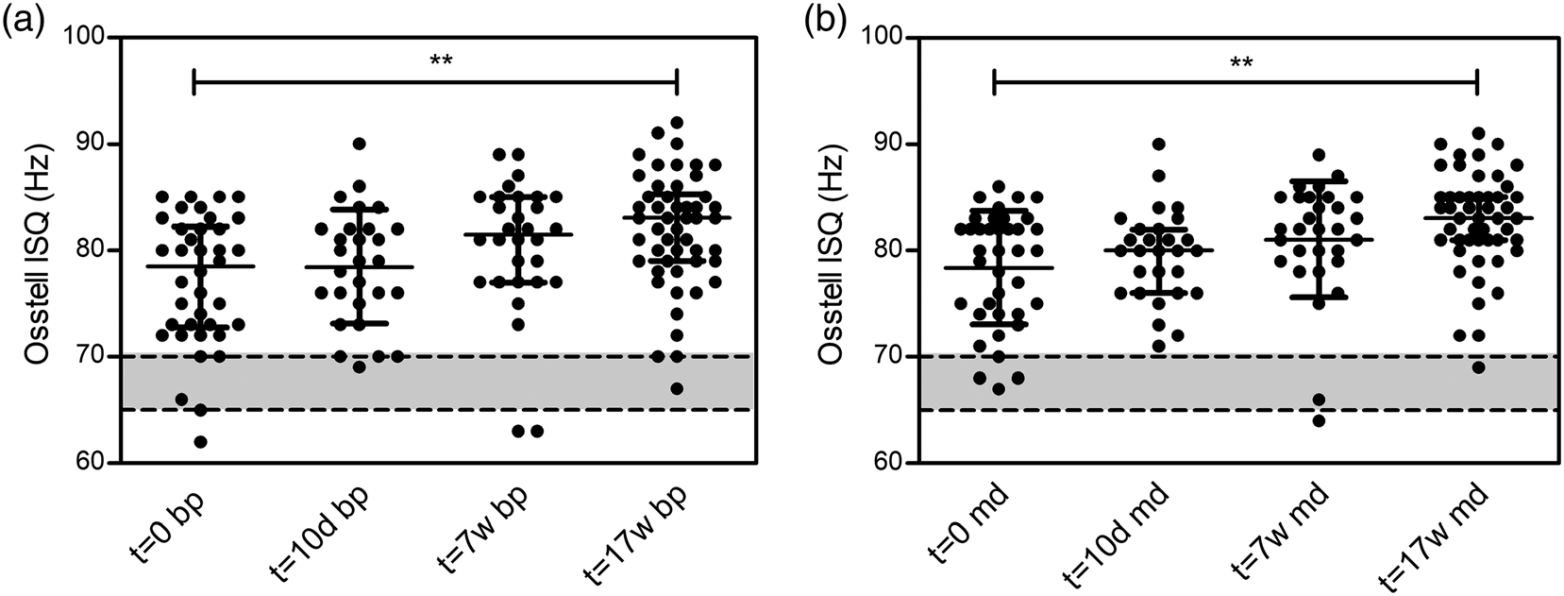
Change in ISQ values during 17‐week follow‐up after implant placement. Whiskers represent mean and corresponding *SD* (*t* = 0, *t* = 7w, *t* = 17w) or median and interquartile range (*t* = 10d) for bp (panel a) and md (panel b) ISQs. Gray area indicates reference value for ISQ. ^**^, *p* < .01 in Kruskal–Wallis test and post hoc comparison with Bonferroni correction. bp, buccal/palatal; d, days; md, mesial/distal; w, weeks

Implant stability, measured as the ISQ, was determined at several time points over the course of the study (Table 1 and Figure 1a,b). Directly after placement of the implant (*t* = 0), median ISQs were bp 73.5 (IQR 5.0) and md 74.5 (IQR 5.0). After 10 days, ISQs were 78.5 (*SD* 5.4) bp and 79.6 (*SD* 4.3) md. The implant stability increased slightly more during the remaining follow‐up, with median bp ISQs of 77.0 (IQR 7.0) and 79.0 (IQR 9.0) and md ISQs of 79.0 (IQR 5.0) and 81.5 (IQR 8.0) at 7 and 17 weeks after implantation, respectively. Together, these data confirm the good primary and secondary stability of the dental implants.

### 3.2 Effect of peripheral blood cell composition on PRF membrane

**Figure 2:**
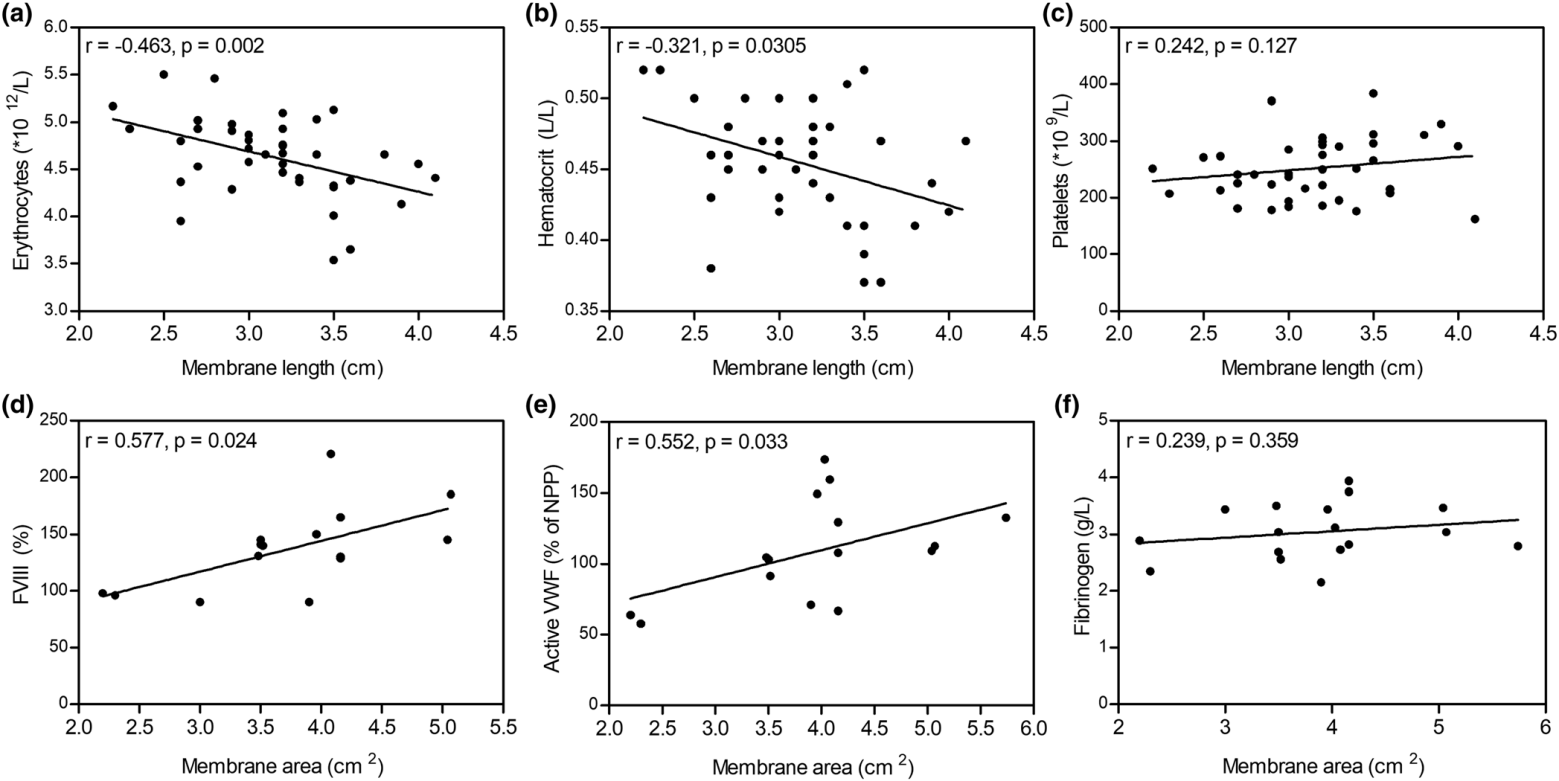
Effects of peripheral blood cells and coagulation factors on PRF membrane. PRF membrane size correlated significantly with (a) erythrocyte count and (b) hematocrit but not with (c) platelet count. Of the coagulation factors, PRF membrane area correlated with (d) FVIII and (e) active VWF levels but not with (f) fibrinogen levels. Spearman correlation coefficients and corresponding *p* values are indicated in the upper left corner. PRF, platelet‐rich fibrin; VWF, von Willebrand factor

We hypothesized that peripheral blood cell composition and coagulation factor levels may influence PRF membrane characteristics (Table 2). A significant inverse correlation between erythrocyte count and PRF membrane length (*n* = 41) was observed: PRF membranes prepared from patients with a higher erythrocyte count in peripheral blood (and higher hematocrit) resulted in shorter PRF membranes than patients with a lower erythrocyte count (Figure 2a,b). Conversely, there were no significant associations between platelets (Figure 2c) and PRF membrane size parameters. FVIII and active VWF levels correlated significantly (*p* = .024 and *p* = .033, respectively) with PRF membrane area (*n* = 17; Figure 2d,e), whereas fibrinogen levels did not correlate with PRF membrane area (Figure 2f) or length.

**Table 2:** Blood cells and coagulation factors in PRF‐treated patients prior to extraction

Variable	N	Mean ± SD OR Median (IQR)	Reference values
Blood cells			
Platelets (×10^9^/L)	50	254.7 ± 55.3	150–400
Erythrocytes (×10^12^/L)	50	4.7 ± 0.5 (Total)	
		4.9 ± 0.4 (Men)	4.4–5.8 (Men)
		4.4 ± 0.3 (Women)	4.0–5.3 (Women)
Leukocytes (×10^9^/L)	50	6.9 (2.5)	4–10
Coagulation factors			
Fibrinogen (g/L)	41	2.9 ± 0.5	2.0–4.0
FXIII (%)	38	83.5 (18)	70–140
Active VWF (% of NPP)	38	126.1 (75.7)	91.6–154.8†
Total VWF (%)	38	99.6 (47.6)	50–150

*Note.* All reference values were derived from the Dutch Society for Clinical Chemistry (“Nederlandse Vereniging voor Klinische Chemie NVKC. Algemeen overzicht referentiewaarden,” 2018) except those indicated by †, which were derived from (van der Vorm et al., 2019).

Abbreviations: NPP, normal pooled plasma; VWF, von Willebrand factor.

### 3.3 Effect of peripheral blood cell composition on implant stability

**Figure 3:**
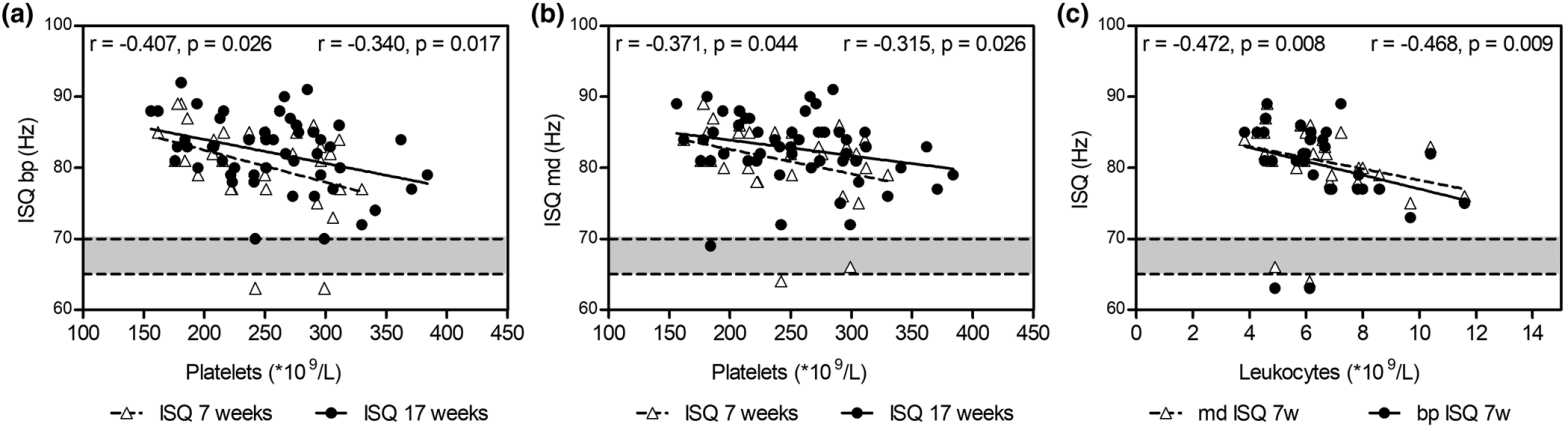
Effects of peripheral blood cells on implant stability*.* Platelet (a, b) and leukocyte (c) counts correlate with implant stability after 7 and 17 weeks. Spearman correlation coefficients and corresponding *p* values are indicated in the upper left corner for the parameter indicated with open triangles and in the upper right corner for parameters indicated with closed circles. Gray area indicates reference value for ISQ. bp, buccal/palatal; ISQ, implant stability quotient; md, mesial/distal

Because we found that specific cellular and coagulation parameters influence PRF characteristics, we were interested in the effect of the composition of peripheral blood on implant stability. Platelet count was significantly inversely correlated with bp (Figure 3a) and md (Figure 3b) ISQ scores at 7 and 17 weeks, whereas erythrocytes were not significantly correlated with implant stability. At 7 weeks postimplantation, higher implant stability was associated with lower leukocyte count (Figure 3c), but this was not observed at 17 weeks.

**Figure 4:**
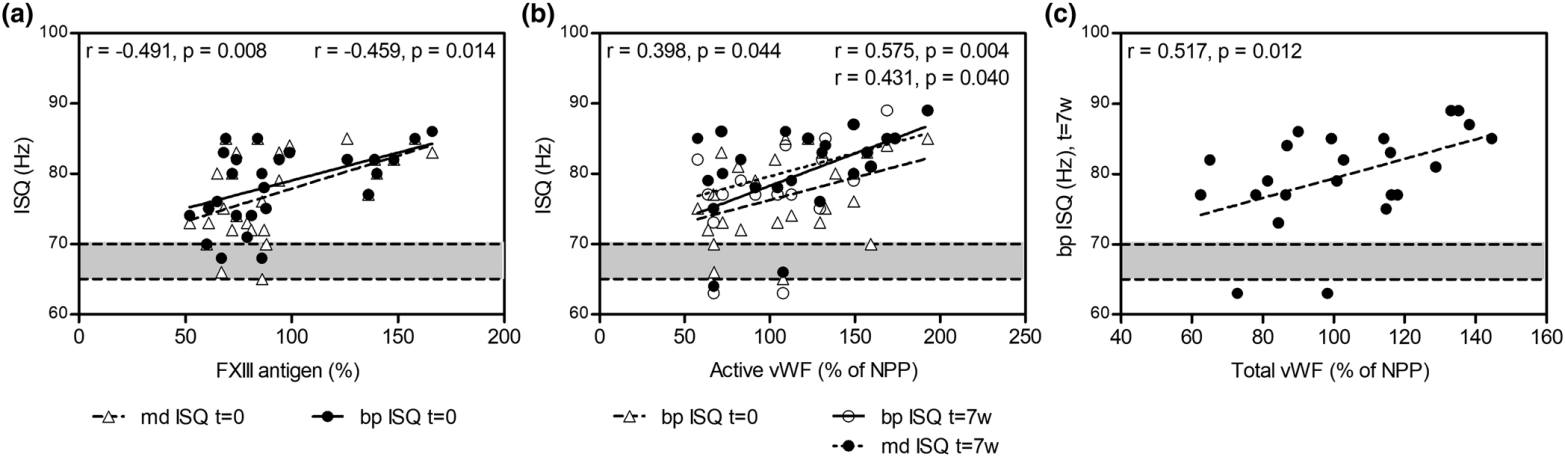
Effects of coagulation factor levels on implant stability. Levels of the coagulation factors FXIII (a), active VWF (b), and total VWF (c) correlate with implant stability after 0 and/or 7 weeks. Spearman correlation coefficients and corresponding *p* values are indicated in the upper left corner for the parameter indicated with open triangles and in the upper right corner for parameters indicated with closed circles (top for open circles and bottom for closed circles in Figure 4b). bp, buccal/palatal; md, mesial/distal; NPP, normal pooled plasma; VWF, von Willebrand factor; w, weeks

Significant correlations between implant stability and a number of coagulation parameters were observed, namely, between ISQ at *t* = 0 and FXIII (Figure 4a) and active VWF (only bp, Figure 4b) and between ISQ at *t* = 7 weeks and active VWF (Figure 4b) and total VWF (only bp, Figure 4c).

## 4 DISCUSSION

In the present study, we analyzed the stability of nonimmediate implants placed following ridge preservation with a mixture of autologous PRF and DBBM xenograft. We found several interesting correlations between implant stability, peripheral blood cell counts, and the coagulation factors (active) VWF and FXIII.

Blood composition may have dual effects on wound healing and tissue regeneration, namely, a direct effect on the composition of the (PRF) clot and an indirect effect on the circulation of the wound area. Conversely, the underlying condition requiring tooth extraction (e.g., periodontitis) may influence peripheral blood cell composition and coagulation status. We found several interesting correlations between blood cell counts and coagulation factor levels on the one hand and membrane characteristics and implant stability on the other hand. Surprisingly, platelet counts were not associated with PRF membrane size. However, higher erythrocyte counts in peripheral blood correlated with shorter PRF membranes. This may be explained by the fractional volume of erythrocytes (hematocrit) and plasma: A lower erythrocyte count (and thus hematocrit) is likely accompanied by a larger plasma fraction after centrifugation, which results in a longer clot. Indeed, hematocrit showed a significant inverse correlation with membrane length (*r* = −.321, *p* < .05), in accordance with findings by others (Mazzocca et al., 2012).

Furthermore, membrane area correlated significantly with FVIII and active VWF levels. FVIII and (active) VWF both play a central role in hemostasis, illustrated by the severe bleeding phenotype associated with hemophilia A and von Willebrand disease, respectively. Hemophilia A patients are known to have more irregular fibrin clot structure than healthy individuals, composed of thicker and shorter fibers, and this effect is mediated through reduced thrombin generation (Wolberg, 2007). Although none of our patients suffered from hemophilia, it could be hypothesized that lower FVIII levels affect the fibrin structure in the PRF clot to produce a smaller membrane. Regarding active VWF, one of the key functions of VWF is to tether platelets to the exposed subendothelial collagen after vessel wall damage. Circulating VWF can only exert this function after conversion to its active conformation, which is induced among others by shear stress (Huizinga et al., 2002). In healthy individuals, only a minute amount of VWF circulates in its active conformation, but nevertheless, there is an interindividual variation of around 15% (van der Vorm et al., 2019). During clot formation, functionally active VWF readily incorporates into the fibrin network and subsequently supports platelet adhesion (Miszta et al., 2014). Also, the presence of VWF results in the formation of a less dense fibrin network (Miszta et al., 2014). Thus, during PRF clot formation, higher active VWF levels may also facilitate binding of more platelets and may result in a more loose fibrin network and, hence, a larger membrane area. Of note, no significant correlation between membrane size and fibrinogen levels was observed, probably because fibrinogen circulates in excess amounts and hence does not limit the size of the PRF membrane.

More of clinical interest is the effect of these peripheral blood parameters on implant stability. Surprisingly, we found that peripheral platelet and leukocyte counts were inversely associated with secondary implant stability (at 7 weeks postimplantation). This finding is counterintuitive as both platelets and leukocytes are considered essential for tissue regeneration and osteogenesis by the release of growth factors and by supporting angiogenesis and lymphogenesis (Soloviev et al., 2014). Our findings challenge this view on the cellular fraction of PRF as the key elements responsible for the clinical efficacy. However, future studies quantifying cell counts as well as growth factor and inhibitor levels within the clot itself are required to support these findings. Similar to our findings on membrane size, active VWF was also associated with primary implant stability and both active VWF and total VWF levels correlated with secondary stability at 7 weeks. As mentioned, (active) VWF may contribute to both platelet incorporation in the fibrin mesh and to fibrin network structure (Miszta et al., 2014). Moreover, activated VWF may indirectly affect implant stability by supporting primary wound healing (Miszta et al., 2014). Levels of FXIII, the coagulation factor responsible for fibrin crosslinking, also correlated with primary implant stability. FXIII is known to be involved in wound healing, as demonstrated by impaired wound repair in FXIII deficient mice (Inbal et al., 2005). Thus, circulating FXIII levels may both directly (through fibrin crosslinking) and indirectly (through wound healing) affect implant stability. Altogether, our data suggest that the blood composition and the fibrin structure of PRF may be critical modulators of implant stability and require more emphasis in the future, more detailed studies.

The most important limitation of our study is the lack of randomization to a PRF treatment group and a control group treated with only DBBM, or alternatively a split‐mouth model with these two treatments. Given the clinical success of implants placed with PRF and DBBM as a grafting material (as observed by the implantologist involved in this study, J. B.), in particular in patients with an infamous prognosis, withholding this treatment from patients to serve as controls for the current study was deemed unethical. As an alternative, we considered including patients with a more favorable prognosis (such as the absence of inflammation and better residual bone at the time of extraction) as a control group treated with only bovine bone substitute. Although this would be a more ethically acceptable approach, it would confer considerable bias to the effect size of the PRF. A second limitation is that we could not determine blood cell count and coagulation factors in the PRF itself. Previously, methods for measurement or estimation of platelet counts in PRF have been proposed, such as the “subtraction method” (Aggarwal, 2015; Dohan Ehrenfest, Del Corso, Diss, Mouhyi, & Charrier, 2010; Eren, Gurkan, Atmaca, Donmez, & Atilla, 2016), but unfortunately, we did not perform these measurements in the liquid fractions after clot formation. The associations between peripheral blood composition and outcomes (membrane size and implant stability) should therefore be interpreted with caution, as the composition of the PRF may be altered relative to the peripheral blood composition. Third, it was not always possible to schedule patients at each of the three time points, explaining that the number of observations is not 50 for all time points and parameters. A future study should take into account this larger than expected loss‐to‐follow‐up rate. Finally, bone quantity around the implants could not be evaluated histologically and correlated with implant stability. Primary stability measurements were previously demonstrated to correlate significantly with bone density (Molly, 2006). However, the lack of a control group in the current study already precludes drawing conclusions on the effect of the combination graft of PRF and DBBM on bone density and implant stability; hence, in further studies, both a control group and histological analysis should be included.

In addition, we are fully aware that association is not the same as causation and that a large variety of patient‐related factors can potentially confound the observed correlations between hematological parameters and implant stability. For instance, age is known to influence coagulation, and older age is associated with higher VWF levels (Favaloro et al., 2005). Although our inclusion criterium was to include all patients >18 years, the majority (43/50, 86%) of our patients was older than 50 years. This may explain why we see no significant associations between age and implant stability itself nor with any of the hematological parameters associated with implant stability. Furthermore, implant stability was not significantly different between men and women or between smokers and nonsmokers. Although the negative effects of smoking on implant stability have been described extensively in literature (Kasat & Ladda, 2012), the number of smokers in the current study is likely too small to detect a significant difference. Likewise, subgroups with comorbidities were too small to perform meaningful statistical analysis. Larger studies are required to assess possible confounding effects of these patient‐related factors on implant stability.

In conclusion, implant stability, following alveolar ridge preservation with PRF and bovine bone substitute, is associated with several hematological parameters. Our results suggest that fibrin structure and levels of (active) VWF and FXIII, more than platelet and leukocyte count, may be determining factors for PRF membrane characteristics and implant stability.

## CONFLICT OF INTEREST

The authors state no conflict of interest.
